# Effect of sample processing and time delay on cell count and chemistry tests in cerebrospinal fluid collected from drainage systems

**DOI:** 10.11613/BM.2018.030705

**Published:** 2018-10-15

**Authors:** Ana Mlinarić, Željka Vogrinc, Zrinka Drenšek

**Affiliations:** 1 Department of Laboratory Diagnostics, University Hospital Centre Zagreb, Zagreb, Croatia; 2 Faculty of Pharmacy and Biochemistry, University of Zagreb, Zagreb, Croatia

**Keywords:** cerebrospinal fluid, extra-ventricular drainage system, cell count, biochemistry analyses, turnaround time

## Abstract

**Introduction:**

Cerebrospinal fluid (CSF) from extra-ventricular drainage (EVD) systems is routinely analysed to diagnose EVD–related bacterial meningitis. We investigated the effect of time delay and sample processing on cell count and basic biochemistry results in EVD CSF to define optimal turnaround time and whether manual and automated cell counting are comparable in such samples.

**Materials and methods:**

In total, 32 EVD CSF samples were analysed. Baseline testing included cell counting (Fuchs-Rosenthal chamber and Sysmex XE5000) and biochemistry analyses (glucose, lactate, proteins). Manual cell counting was also performed at intervals of 61-90 and 91-150 minutes from baseline in the residual sample. Biochemistry analyses were performed in samples before and after centrifugation at baseline and at 91-150 minutes interval.

**Results:**

At 91-150 minutes total cell count (P < 0.001), large lymphocytes (P = 0.007), neutrophils (P < 0.001) and phagocytes (P = 0.006) obtained by manual counting decreased and the number of disintegrated cells count increased (P = 0.016) compared to the baseline values. Considering method comparison, proportional difference between methods for all cell (sub)groups was obtained, whereas polymorphonuclears also showed the constant difference (y = 11.21 + 1.22x). Compared to centrifuged CSF, lower concentration of glucose and lactates were obtained in uncentrifuged samples (P < 0.001) at baseline.

**Conclusions:**

Manual cell counting should be performed within 60 minutes as any delay can alter results. The same counting technique should be used to obtain longitudinally assessable results. Biochemistry tests are stable in uncentrifuged CSF up to 2.5 hours.

## Introduction

Cerebrospinal fluid (CSF) assays are an integral part of diagnostics of neurological diseases, especially in the emergency neurological patients. In such cases, it is imperative that the results are available as soon as possible to assist in diagnosis and further patient treatment.

Extraventricular drainage (EVD) system is indicated for the management of patients who suffered subarachnoid or intracranial haemorrhage, hydrocephalus, shunt malfunction or have a tumour (*1*, *2*). Such patients have a higher risk of associated central nervous system (CNS) infection (*1*, *3*, *4*). As clinical signs are not indicative of CNS infection and because complete neurological evaluation is impossible in ventilated and sedated patients, sampling from EVD system and laboratory analysis of CSF is often performed to diagnose external drainage–related bacterial meningitis at an early stage (*5*).

In patients with bacterial meningitis without EVD system, CSF pleocytosis, low glucose, elevated lactate and proteins are highly indicative of the disease (*1*, *6*). However, in patients with EVD systems, the composition of CSF is different due to the underlying condition or the effects of neurosurgical processes (*1*, *7*). Although CSF culture remains to be the gold standard in the diagnosis of bacterial meningitis, numerous studies have evaluated the potential predictive value of CSF routine laboratory tests for predicting EVD associated ventriculitis (*2*, *3*, *5*, *7*-*10*).

Clinical and Laboratory Standards Institute (CLSI) H56-A guidelines on analysis of cellular components in body fluids recommend that cells should be natively counted using a counting chamber. Cell counting should be performed as soon as possible because of rapid *in vitro* decomposition of cells (*11*). Furthermore, metabolites, such as glucose and lactate, are highly dependent on *in vitro* metabolic processes (*12*).

According to the consensus protocol for the standardization of CSF collection, the CSF samples obtained by lumbar puncture (LP) should be processed as soon as possible but the document also acknowledges that processing of CSF samples within an hour is not a common practice in most laboratories. Thus, the main recommendation is that sample processing should be done within a time delay of 1.5 hours (± 30 minutes) (*13*). At present, there are no recommendations about optimal processing of the EVD samples. Samples of CSF from EVD system are partially different from lumbar CSF samples because they often contain a high number of various cells and cell debris. Therefore, manual counting can be tedious and delay the reporting of results (*14*, *15*). For these reasons, the automated method for cell counting would be preferable to manual counting, but there are no recommendations whether both methods can be used simultaneously without restriction for samples collected from EVDs. Furthermore, processing CSF samples before analysis inevitably postpone results reporting due to the time needed for centrifugation.

The aim of this study was to investigate the effect of time delay and sample processing on cell count and basic biochemistry results in CSF obtained from EVD systems to define optimal turnaround time (TAT) for these samples. Additionally, we have compared automated and manual counting methods to investigate whether both methods could be performed interchangeably in EVD samples.

## Materials and methods

### Materials

This prospective experimental study was performed from October 2016 to January 2017 in Department of Laboratory Diagnostics, University Hospital Centre Zagreb, Croatia. Consecutive CSF samples collected from EVD systems using standard collection procedure of adult patients from neurological intensive care units, and delivered to the laboratory for routine processing in the morning hours (from 7 to 11 am) with sufficient sample volume (> 2 mL) were included (*16*). In total, we have analysed 32 CSF samples collected from EVD. Extremes in cell count measurement were identified visually by scatter plot analysis if values were unevenly distributed across the concentration range. Two samples were therefore excluded from further cell count method comparison analysis as these samples had extremely high cell number, which would influence Passing-Bablok regression analysis. All samples were anonymous samples collected for standard laboratory practice and all testing regarding sample stability was performed on residual samples. No additional sample was collected specifically for this study.

For this type of study, informed consent is implied and ethical committee approval is not necessary. The study was performed according to the requirements of the Declaration of Helsinki.

### Methods

**Figure 1:**
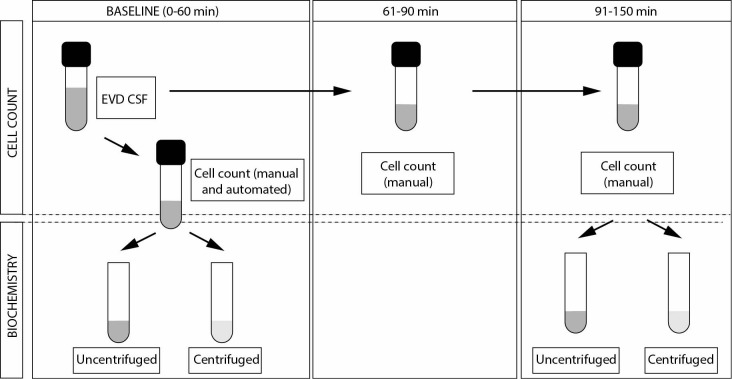
Schematic representation of the study design. Upon sample admission, EVD CSF was thoroughly mixed to achieve a homogenized sample. Routine testing of CSF included manual cell counting and biochemistry analyses in centrifuged sample. Additionally, automated cell counting and biochemistry analysis in uncentrifuged sample were performed. These results were used as baseline values for this study. The residual materials of EVD CSF were used for testing the effect of time delay and sample processing on cell count and biochemistry tests. The tube with uncentrifuged residual sample was kept closed at room temperature (range 22-26 °C) and was used for manual cell counting at 61-90 min and 91-150 min and for biochemistry analyses in uncentrifuged and centrifuged sample. Measurements included: cell counting (white blood cell count, red blood cell count, polymorphonuclear cells, mononuclear cells) and glucose, lactate and total proteins. CSF - cerebrospinal fluid. EVD - extraventricular drainage.

Upon sample admission, EVD CSF was thoroughly mixed to achieve a homogenized sample. Routine testing of CSF included manual cell counting and biochemistry analyses in centrifuged sample. Additionally, automated cell counting and biochemistry analysis in uncentrifuged sample were performed. These results were used as baseline values for this study. The residual materials of EVD CSF were used for testing the effect of time delay and sample processing on manual cell count and biochemistry tests (Figure 1). The tube with uncentrifuged residual sample was kept closed at room temperature (range 22-26 °C) and was used for manual cell counting at 61-90 min and 91-150 min and for biochemistry analyses. Due to the limited sample volume, biochemistry analyses were performed only in the time delay of 91-150 min, in both uncentrifuged and centrifuged aliquots (Figure 1). Samples were centrifuged on an MPW-223e centrifuge (MPW Med. Instruments, Warsaw, Poland) for 10 minutes at 2000xg.

#### Cell counting

At the baseline, the EVD CSF cell count was performed by two methods: manual and automated. Firstly, the total cells (TC) and red blood cells (RBC) were counted in native CSF using the Fuchs-Rosenthal chamber. Total cells were differentiated to small and large lymphocytes, neutrophil granulocytes, phagocytes and disintegrated cells and results were expressed as a cell number/3x10^6^/L (*17*). Counting was performed using Olympus BX41 light microscope (Olympus, Tokyo, Japan) under the 400x magnification.

The cell count was also performed in Body fluid (BF) mode on the Sysmex XE5000 haematology analyser (Sysmex Corporation, Kobe, Japan). The cell count in BF mode included: white blood cells (WBC), red blood cells (RBC), polymorphonuclear cells (PMN) and mononuclear cells (MN). Because of the limited sample volume, counting in Body fluid mode was not repeated at delayed time intervals. White blood cells parameter corresponds to TC in the manual count. The results were expressed as a cell number/3 x10^6^/L.

#### Glucose, lactate and proteins measurement in CSF

Glucose, lactate and total protein concentrations were measured on Cobas 6000 c501 biochemistry analyser (Roche Diagnostics GmbH, Mannheim, Germany) with standard methods, using reagents GLUC3 (enzymatic reference method with hexokinase), LACT2 (enzymatic method with lactate oxidase) and TPUC3 (precipitating method with benzethonium chloride), respectively (Roche Diagnostics GmbH, Mannheim, Germany). For each analyte, quality control was performed daily in two levels using PreciControl ClinChem Multi 1 and PreciControl ClinChem Multi 2 (Roche Diagnostics GmbH, Mannheim, Germany), and Precinorm PUC and Precipath PUC controls (Roche Diagnostics GmbH, Mannheim, Germany). Coefficients of variations in the tested time period were within analytical requirements for imprecision.

### Statistical analysis

Data were tested for normality using the Shapiro-Wilk test. Data did not follow a normal distribution and were presented as median (interquartile range (IQR)). Statistical differences were tested using the Wilcoxon rank sum test for paired samples. Spearman correlation was performed as a *post-hoc* test to test whether the differences in biochemistry tests between centrifuged and uncentrifuged CSF are associated with the TC or RBC count. Comparisons of automated and manual cell counting methods were performed using Passing-Bablok regression, presented with regression equation and 95% confidence intervals (95% CI) for slope and intercept, and Bland Altman analysis. The following cell subgroups were compared: WBC and TC, PMN and neutrophil granulocytes, MN and the sum of small and large lymphocytes. A P value < 0.05 was set as a level of statistical significance. Statistical analysis was performed using MedCalc Software v.17.4.4 (Ostendt, Belgium).

## Results

A total number of 32 CSF samples collected from EVD system were included in the study. Median (IQR) of tested analytes at each time interval and sample processing status are summarized in Tables 1 and 2.

**Table 1:** Values of manual cell counting in cerebrospinal fluid collected from extraventricular drainage systems (N = 32) at the baseline and delayed time intervals

	**Time interval of analysis**				
**CSF cells (/3 x10^6^/L)**	**0-60 minutes (baseline)**	**61-90 minutes**	P*	**91-150 minutes**	**P^†^**
**Total cell number**	199 (82 - 573)	193 (82 - 583)	0.033	183 (56 - 581)	< 0.001
**Small lymphocytes**	17 (4 - 132)	9 (3 - 114)	0.265	13 (2 - 124)	0.167
**Large lymphocytes**	14 (5 - 36)	13 (4 - 43)	0.166	13 (4 - 33)	0.007
**Neutrophil granulocytes**	72 (8 - 495)	71 (9 - 498)	0.046	49 (12 - 496)	< 0.001
**Phagocytes**	2 (0 - 5)	1 (0 - 6)	0.020	1 (0 - 4)	0.006
**Disintegrated cells**	7 (1 - 20)	7 (3 - 20)	1.000	10 (3 - 34)	0.016
**Red blood cells**	3630 (184 -13,055)	2990 (165 - 14,646)	0.923	3140 (149 - 13,415)	0.202
Values are presented as median (interquartile range). *Difference between baseline and 61-90 minutes time delay. ^†^Difference between baseline and 91-150 minutes time delay. P < 0.05 was considered statistically significant.					

**Table 2:** The results of biochemistry analyses in uncentrifuged and centrifuged cerebrospinal fluid from extraventricular drainages at baseline and with the time delay

	**Time interval of analysis**						
	**0-60 min (baseline)**			**91-150 min**			
**Analyte (units)**	**Uncentrifuged**	**Centrifuged**	P*	**Uncentrifuged**	**Centrifuged**	**P^†^**	**P^‡^**
**Glucose (mmol/L)**	3.63 (2.60 - 4.07)	3.70 (2.67 - 4.32)	< 0.001	3.69 (2.67 - 4.12)	3.78 (2.69 - 4.33)	0.006	0.797
**Lactate (mmol/L)**	3.24 (2.37 - 4.37)	3.48 (2.46 - 4.76)	< 0.001	3.18 (2.42 - 4.16)	3.25 (2.48 - 4.79)	< 0.001	0.493
**Total proteins (g/L)**	0.71 (0.41 - 1.49)	0.68 (0.43 - 1.62)	0.249	0.69 (0.37 - 1.55)	0.65 (0.39 - 1.62)	0.316	0.866
Values are presented as median (interquartile range). *Difference between uncentrifuged and centrifuged samples at the baseline. ^†^Difference between uncentrifuged and centrifuged samples 91-150 minutes time delay. ^‡^Difference of centrifuged sample at the baseline and centrifuged sample at 91-150 minutes time delay. P < 0.05 was considered statistically significant.							

Throughout the tested period regarding the manual counting, the number of RBCs (P = 0.202) and small lymphocytes (P = 0.167) remained unchanged compared to the baseline values. Already, at 61-90 minutes interval, TC count (P = 0.033), neutrophil granulocytes (P = 0.046) and phagocytes (P = 0.020) decreased compared to the baseline counting. Finally, at 91-150 minutes interval, large lymphocytes decreased (P = 0.007) and the number of disintegrated cells increased (P = 0.016) compared to the baseline count (Table 1).

**Figure 2:**
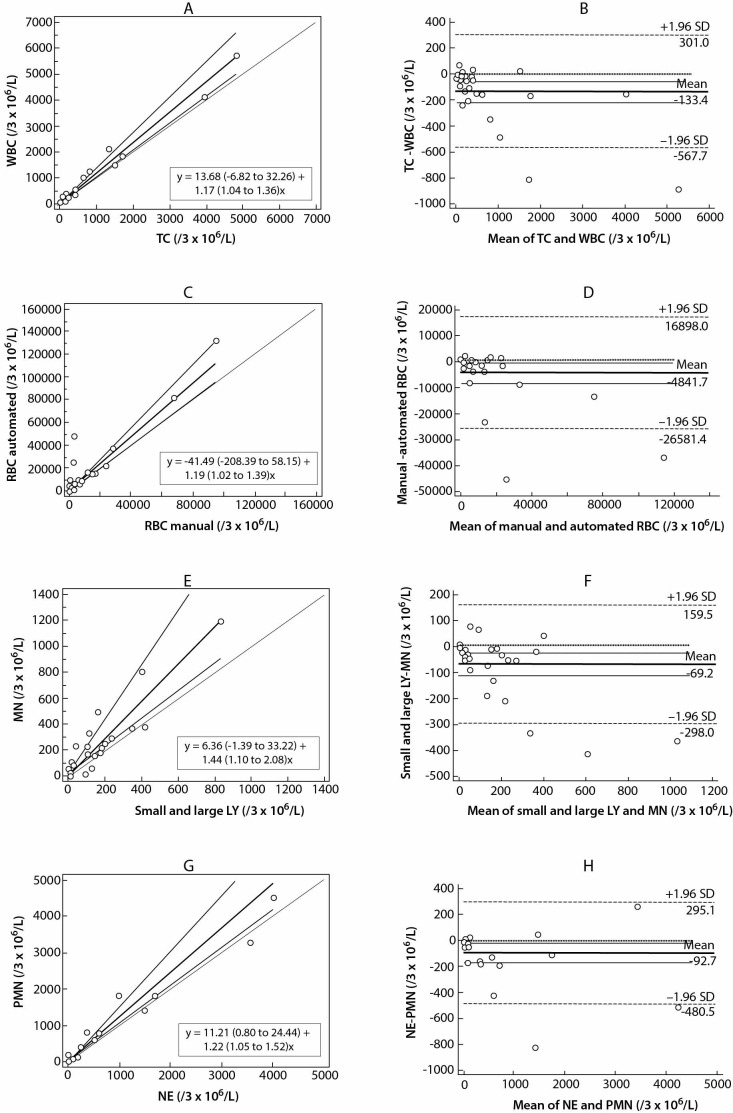
The comparison results of automated and manual method of cell counting in cerebrospinal fluid collected from extraventricular drainage systems. The following cell subgroups from automated and manual method, respectively, were compared using Pasing Bablok regression analysis and Bland Altman graph: WBC and TC (A,B); RBC from automated and manual counting (C,D); MN and the sum of small and large lymphocytes (E,F); PMN and neutrophil granulocytes (G,H). The analysis shows the proportional difference in WBC, RBC and MN, while PMN have both proportional and constant difference. Automated method significantly overestimates the results. Passing Bablok regression line (solid line), the confidence interval for the regression line (dashed lines) and identity line (x=y, dotted line) are shown. Bland Altman graph: mean difference (solid line), 95% confidence interval of mean difference (dashed and dotted line), ± 1.96 standard deviations of mean difference (dashed line), identity line (dotted line). WBC - white blood cells. TC - manual total cell count. RBC - red blood cells. MN - mononuclear cells. PMN - polymorphonuclear cells. NE - neutrophil granulocytes. LY - lymphocytes.

In a comparison of the automated and manual method of cell counting, two EVD CSF samples were excluded from analysis: a sample with WBCs 17,760/3 x10^6^/L and a sample with 632,320/3 x10^6^/L RBCs. These samples had extremely high cell counts, which would influence Passing-Bablok regression analysis. Automated and manual methods for cell counting showed: the proportional difference for WBC [y = 13.68 (95% CI: - 6.82 to 32.26) + 1.17 (95% CI: 1.04 to 1.36)x, RBCs [y = - 41.49 (95% CI: - 208.39 to 58.15) + 1.19 (95% CI: 1.02 to 1.39)x and MN [y = 6.36 (95% CI: - 1.39 to 33.22) + 1.44 (95% CI: 1.10 to 2.08)x, while PMN have both proportional and constant difference [y = 11.21 (95% CI: 0.80 to 24.44) + 1.22 (95% CI: 1.05 to 1.52)x] (Figure 2). Bland Altman analysis shows that measurements obtained by an automated method are statistically significantly higher compared to the manual count. Mean bias of automated method for WBC, RBC, MN and PMN are 133/3x10^6^/L 4842/3 x10^6^/L, 69/3 x10^6^/L and 93/3 x10^6^/L, respectively. Automated method always overestimated the results compared to manual counting.

The results of biochemical tests in uncentrifuged EVD CSF samples were available very quickly with a median time of 16 min (IQR 6-28) from sample admission. Median time to results was 27 min (IQR 22-37) for centrifuged samples. However, compared to the centrifuged EVD CSF samples assayed within 60 minutes of sample admission, uncentrifuged samples had lower glucose (P < 0.001) and lactate (P < 0.001) concentration. Glucose (P = 0.006) and lactate (P < 0.001) concentrations were also lower in uncentrifuged CSF samples compared to centrifuged samples at the 91-150 minutes interval (Table 2). Differences in biochemistry tests between uncentrifuged and centrifuged tubes did not correlate with cell content (Table 3). Total protein concentration remained unchanged irrespective of the time delay (P = 0.866) and sample processing status (P = 0.249). The time delay in sample centrifugation had no statistically significant effect on glucose and lactate concentrations (P = 0.797 and 0.493, respectively) (Table 2).

**Table 3:** Correlation of manual total cell count and red blood cells count and the difference in concentrations of glucose and lactate in uncentrifuged and centrifuged extraventricular drainage samples at different time intervals

	Δ* Glucose				Δ* Lactate			
	**0-60 min**		**91-150 min**		**0-60 min**		**91-150 min**	
**Analyte**	**r**	**P^†^**	**r**	**P^†^**	**r**	**P^†^**	**r**	**P^†^**
Total cell count	0.07	0.718	0.02	0.930	0.05	0.807	0.23	0.213
Red blood cells	0.29	0.106	0.14	0.449	0.25	0.163	0.09	0.636
*Δ = concentration (uncentrifuged EVD sample) - concentration (centrifuged EVD sample). ^†^Spearman correlation test was performed, correlation coefficient (r) and corresponding P value are presented. EVD - extraventricular drainage. P < 0.05 was considered statistically significant.								

## Discussion

Cerebrospinal fluid samples from EVD system are partially different from CSF samples obtained by lumbar puncture and represent a special category of biological material. The stability of different analytes in such samples is not well established. We investigated the effect of time delay and sample processing on cell count and basic biochemistry results in these samples.

Our results showed that a relatively short delay in cell counting (61-90 minutes) caused a statistically significant fall in total cell count and granulocyte count by manual method. These results are in accordance with previously reported results for CSF obtained by LP that indicated that the delay in WBC counting could produce false negative results in 40% of samples with pleocytosis and as high as in 86% of the samples with a mild elevation in WBC count (*18*). Also, the selective loss of granulocytes was observed thereby possibly impacting clinical decision (*18*). Therefore, we strongly recommend processing the CSF samples from EVD system within a time delay of maximum 60 minutes.

Manufacturer recommends measuring glucose in CSF immediately as such samples can be contaminated with bacteria and can often contain other cellular constituents. Otherwise, storing the sample at 4 °C or - 20 °C is recommended (*19*). During the routine laboratory work delay in sample processing is likely and in such scenario, the sample would probably be left at room temperature until analysis. We found that glucose, lactate and proteins in EVD samples are stable even when the centrifugation is delayed for 2.5 hours and despite a relatively high cell number in most samples. Other published literature confirms our results. A study by Dujmovic and Deisenhammer proved glucose and lactate in centrifuged CSF samples are stable for 4 hours, at 4 °C (*20*). Results of an *in vitro* study suggest that the effect of RBC contribution on lactate concentration becomes significant only after 6 hours (*21*). Furthermore, the manufacturer declares lactate stability in CSF for 3 hours at room temperature and proteins one day at 15 - 25 °C (*22*, *23*). The stability of lactate and proteins in EVD samples up to 2.5 hours was confirmed in our study.

Analysing uncentrifuged EVD CSF samples is unacceptable because glucose and lactate concentrations were significantly lower compared to centrifuged samples. This effect was present regardless of time delay and was not associated with total cells or red blood cells count. Therefore, faster TAT cannot be achieved by omitting centrifugation, even in samples with low cell counts.

Due to increased use of automated methods for cell counting in CSF, especially in emergency services, we analysed EVD samples at the baseline by both manual and automated methods, to establish if they could be used simultaneously and/or as a substitute for each other. Contrary to previously reported studies comparing CSF samples collected by lumbar puncture, an automated and manual method for cell counting proved not to be comparable when assaying CSF collected from EVD in our study (*15*, *24*–*27*). The reason for this disagreement could be the interference of cell debris in such samples. One other study reported the results of the comparison of Sysmex XE 5000 and manual counting using ventricular samples collected from EVD which showed both proportional and constant difference between methods, with some highly discrepant findings (*14*). These authors argue that despite discrepant results due to interferences, such high WBC counts are still indicative of infection (*1*, *5*, *9*, *28*). We were unable to find clinical decision limits for this material in the published literature. For this reason we did not discuss the clinical significance of the method bias. Several studies propose that the increases in the daily CSF cell count are highly indicative of bacterial infection (*5*, *29*). Therefore, patients are followed longitudinally and CSF parameters and clinical status combinedly assessed. In such cases, the high comparability of test results is especially important. Accordingly, the method should always be stated in the laboratory report if automated and manual counting methods are used interchangeably in routine practice for EVD CSF samples.

Limitations of the present study include small sample size and uneven distribution of the measurements that could influence the comparison of the manual and automated methods of cell counting in EVD samples. This could also be the reason for the disagreement with the results of other studies performed in CSF collected by lumbar puncture. However, due to the difficulty in sampling CSF from EVD, limited amount of the collected material, and the relatively long duration of the study, we consider that the included sample size is representative for the studied population. Furthermore, the information on time passed from the sampling to the laboratory was not available, and we were only able to investigate the effect of time delay in EVD samples within the laboratory. Due to the insufficient CSF volume, we were unable to test the effect of time delay in more time intervals. Therefore, we have limited our study design to test only the effect of 2.5 hours time delay on biochemistry analytes and compared automated and manual cell counting only at the baseline.

In summary, our results confirmed that delay in cell counting in EVD CSF samples causes a significant fall in total cell count and granulocyte count. Therefore, we recommend TAT for cell number analysis of maximum 60 minutes. Glucose, lactate and total proteins are stable in uncentrifuged EVD CSF samples meaning the eventual delay in sample processing would not influence analytical results, but samples have to be centrifuged prior to biochemistry analysis. Results of manual and automated counting methods are not comparable for EVD samples. It is therefore imperative to always use the same counting technique to obtain results which could be correctly longitudinally assessed.
